# The Odorant Binding Protein Gene Family from the Genome of Silkworm, *Bombyx mori*

**DOI:** 10.1186/1471-2164-10-332

**Published:** 2009-07-23

**Authors:** Da-Ping Gong, Hui-Jie Zhang, Ping Zhao, Qing-You Xia, Zhong-Huai Xiang

**Affiliations:** 1 The Key Sericultural Laboratory of Agricultural Ministry, Southwest University, Chongqing, PR China; 2 Key Laboratory for Tobacco Quality Control, Ministry of Agriculture, Tobacco Research Institute, Chinese Academy of Agricultural Science, Qingdao, PR China; 3 Institute of Agronomy and Life Sciences, Chongqing University, Chongqing, PR China

## Abstract

**Background:**

Chemosensory systems play key roles in the survival and reproductive success of insects. Insect chemoreception is mediated by two large and diverse gene superfamilies, chemoreceptors and odorant binding proteins (OBPs). OBPs are believed to transport hydrophobic odorants from the environment to the olfactory receptors.

**Results:**

We identified a family of OBP-like genes in the silkworm genome and characterized their expression using oligonucleotide microarrays. A total of forty-four OBP genes were annotated, a number comparable to the 57 OBPs known from *Anopheles gambiae *and 51 from *Drosophila melanogaster*. As seen in other fully sequenced insect genomes, most silkworm OBP genes are present in large clusters. We defined six subfamilies of OBPs, each of which shows lineage-specific expansion and diversification. EST data and OBP expression profiles from multiple larvae tissues of day three fifth instars demonstrated that many OBPs are expressed in chemosensory-specific tissues although some OBPs are expressed ubiquitously and others exclusively in non-chemosensory tissues. Some atypical OBPs are expressed throughout development. These results reveal that, although many OBPs are chemosensory-specific, others may have more general physiological roles.

**Conclusion:**

Silkworms possess a number of OBPs genes similar to other insects. Their expression profiles suggest that many OBPs may be involved in olfaction and gustation as well as general carriers of hydrophobic molecules. The expansion of OBP gene subfamilies and sequence divergence indicate that the silkworm OBP family acquired functional diversity concurrently with functional constraints. Further investigation of the OBPs of the silkworm could give insights in the roles of OBPs in chemoreception.

## Background

Olfactory and gustatory systems play crucial roles for insects in finding food, mates, and oviposition sites. Exquisitely sensitive chemosensory systems of insects can recognize and discriminate diverse chemicals. Olfaction is mediated by specific olfactory sensory neurons, which project their dendrites into a lymphatic cavity where odorant binding proteins (OBP) are present at high concentrations. It has been suggested that OBPs have key functions in recognizing and delivering hydrophobic odorants to olfactory receptors (OR) on dendritic membranes [1-4].

Insect OBPs are a class of small water-soluble extracellular proteins with molecular masses of approximately 14 kDa [5]. They are believed to serve multiple functions. For example, OBPs may act as solubilizers and carriers of the lipophilic odorants in sensillum lymph and also as semi-selective filters in odorant discrimination. Furthermore, OBPs may present odorants to activate neuronal receptors or to sequester and deactivate odorants after stimulation [3]. Experimental evidence has demonstrated that OBPs could selectively bind odorants or pheromones [6-8]. Silkworm pheromone-binding protein 1 (BmPBP1) is capable of enhancing sensitivity and selectively mediating the response to bombykol rather than bombykal [9,10]. Recently, several studies have shown that OBPs are required for correct recognition of some odors. In *Drosophila*, LUSH mutants are defective for avoidance of concentrated alcohols or benzaldehyde and have complete loss of sensitivity to the pheromone 11-cis vaccenyl acetate [11-13]. LUSH undergoes a pheromone-specific conformational change that triggers the firing of pheromone-sensitive neurons [14]. Two other odorant binding proteins for *Drosophila*, OBP57d and OBP57e, are not only involved in taste perception but can also change the behavioral response to toxins contained in fruit [15]. Four polymorphisms in three OBP genes in the *Obp99 *cluster are associated with variation in olfactory response to benzaldehyde in *Drosophila *[16]. In the fire ant, *Solenopsis invicta*, allelic variantion in pheromone-binding protein GP-9 regulates social organization of colonies. Single-queen colonies are always homozygous for the B allele, whereas multiple-queen colonies possess at least one copy of the b variant [17].

Recent progress in whole-genome sequencing of insects has provided insights into the molecular mechanisms of olfaction. In the dipteran species *D. melanogaster *and *A. gambiae*, more than 50 OBPs and approximately 70 Ors have been identified [18-22]. Subsequently, 21 OBPs and 170 Ors have been annotated in the bee [23,24]. Insects have far fewer ORs but more OBPs than do nematodes and mammals [25]. In mammals, OBPs show fairly broad binding spectra and seem capable of acting only as general carriers [26]. It has been proposed that odorant discrimination in insects might be due to combined usage of ORs and OBPs [19]. Nevertheless, insect OBPs have also been found in non-chemosensory tissues, implicating them in broader non-olfactory functions [27-29].

The silkworm, *Bombyx mori *is an oligophagous, economically important member of the Lepidoptera, a taxonomic group that includes numerous pests of agriculture and forestry. Silkworm is a well-established model for studying insect olfaction [30]. In addition, the olfactory system of the giant sphinx moth *Manduca sexta *has been investigated in depth by the Hildebrand lab [31]. Studies of the role of OBPs in molecular olfaction in silkworm may help us to understand and manipulate olfactory-driven food preference in this and other Lepidoptera. Prior to this study, only four silkworm OBP genes had been reported [32].

Our annotation of forty-four candidate BmorOBP genes in the silkworm genome revealed lineage-specific subfamily expansions in the silkworm OBP family. Based on a genome-wide oligonucleotide microarray, the expression profiles of 32 candidate BmorOBP genes were detected in different tissues of day three 5th instar and through development from fifth instars to adult moths. Some BmorOBPs were specific to olfactory tissues and others were expressed broadly in non-olfactory tissues. We found several OBP genes whose expression patterns were sexually dimorphic.

## Results

### The OBP gene family in the silkworm

We have identified a total of 44 candidate OBP genes in the newly assembled silkworm genome (Table 1). These include the four previously identified OBPs: PBP1 (pheromone binding protein), GOBP1 and GOBP2(general odorant binding proteins), and ABPX (antennal binding protein) [32]. Additionally, the PBP2 and PBP3 identified herein have been deposited in GenBank (accession numbers AM403100 and AM403101). Thirty-five OBP-like genes were predicted by the GLEAN algorithm, 17 of which had their intron-exon boundaries corrected manually. Genomic regions containing another eight OBP genes were predicted by FGENESH+ and exon/intron splice site prediction software. We found many ESTs to support the prediction of 24 of the OBP genes. Because of the poor similarity of the signal peptide sequences, the first exons were difficult to predict for seven of the OBPs. OBP24 was incomplete, lacking the N-terminal region. This gene may be intact in the genome or a pseudogene. OBP9 was identified from EST data, but the entire gene is missing from the current assembly of the genome. It is possible that the current assembly does not cover the entire genome. The corresponding annotations on the Silkworm Genome Database (SilkDB) have been updated .

**Table 1:** The silkworm OBP genes family

New name	Previous name	Scaffold ID	Chromosome	Annotation	Probe	Expression
BmorOBP1	BmorGOBP1	nscaf3052	19	BGIBMGA012611	sw04017	An1
BmorOBP2	BmorGOBP2	nscaf3052	19	BGIBMGA012614	sw15189	An1
BmorOBP3	BmorPBP1	nscaf3052	19	BGIBMGA012615	sw09490	An6
BmorOBP4		nscaf3052	19	BGIBMGA012616*	sw09491	
BmorOBP5		nscaf3052	19			
BmorOBP6		nscaf3052	19	BGIBMGA012617	sw01959	
BmorOBP7		nscaf2902	18	BGIBMGA008356*	sw11831†	
BmorOBP8		nscaf2902	18	BGIBMGA008355*	sw11831†	Mg2
BmorOBP9						Mg2, Ep7
BmorOBP10		nscaf2902	18			
BmorOBP11		nscaf2902	18	BGIBMGA008354	sw22119	Mg100
BmorOBP12		nscaf2902	18			Mg12
BmorOBP13		nscaf2902	18	BGIBMGA008353*	sw15408	Mg3
BmorOBP14		nscaf2902	18		sw15407	An1
BmorOBP15		nscaf2902	18	BGIBMGA008352*	sw21849	
BmorOBP16		nscaf2902	18			
BmorOBP17		nscaf2902	18	BGIBMGA008351		
BmorOBP18		nscaf2902	18	BGIBMGA008474		
BmorOBP19		nscaf2902	18		sw13149†	
BmorOBP20	BmorABPX	nscaf2330	26	BGIBMGA002308	sw00604	
BmorOBP21		nscaf2674	5	BGIBMGA003463*		
BmorOBP22		nscaf2529	5	BGIBMGA002630		Em1
BmorOBP23		nscaf2529	5	BGIBMGA002629	sw03370	Mg37, Sm2, Wd1
BmorOBP24		nscaf2529	5	BGIBMGA002628	sw12016	
BmorOBP25		nscaf2529	5	BGIBMGA002627	sw20121†	
BmorOBP26		nscaf2529	5	BGIBMGA002666*	sw20121†	Mg1
BmorOBP27		nscaf2529	5	BGIBMGA002626	sw20121†	Sp3, Sm3, An3, Mt1, Fs1, Bp1, Mi2, Wd10, Te1
BmorOBP28		scaffold896				
BmorOBP29		nscaf2983	7	BGIBMGA010039*	sw15978	Te2, Fp1
BmorOBP30		nscaf2983	7	BGIBMGA010011*	sw04366†	Bp1
BmorOBP31		nscaf2983	7	BGIBMGA010010	sw15426	Ce2, Bs8, Bp24
BmorOBP32		nscaf2943	14	BGIBMGA009290*	sw00262	Pb1, He1
BmorOBP33		nscaf2943	14	BGIBMGA009291		
BmorOBP34		nscaf2943	14	BGIBMGA009292*		
BmorOBP35		nscaf2943	14			
BmorOBP36		nscaf2948	14	BGIBMGA009352*	sw14581	
BmorOBP37		nscaf2948	14	BGIBMGA009366*	sw05807	
BmorOBP38		nscaf3063	16	BGIBMGA013247	sw14065	Mg4
BmorOBP39		nscaf1681	22	BGIBMGA000225*	sw00870	EP1
BmorOBP40		nscaf3026	23	BGIBMGA011298*	sw11835	Fl2
BmorOBP41		nscaf3026	23	BGIBMGA011276*	sw11149	Ov5, Ep1
BmorOBP42		nscaf3027	23	BGIBMGA011433	sw20434	Mg2
BmorOBP43		nscaf3027	23	BGIBMGA011432*	sw03198	Ov6, Fs3, fl3, fp2
BmorOBP44		nscaf2993	12	BGIBMGA010425	sw09301	Fp3

This table lists the nomenclature, accession numbers, ID, Chromosome, array probe and expression of all 42 odorant binding-like protein genes identified in this study. We have modified annotations to some of the BmOBP genes as indicated by the asterisk. four probes with approximately 20 bases mismatched to the gene sequences are marked with a cross mark. The tissue type of EST data was indicated using abbreviations: Mg (maxillary galea), Sm (middle silkgland), Sp (posterior silkgland), Wd (wing disk), Mt (Malpighian tubule), Fl (Fat body), Mi (Midgut), Te (Testis), He (Hemocyte), Ep (Epidermis) (5th instar larva day 3), Fp (Fat body, pupa 0), Fs (Fat body, spinning 2), Ov (Ovary, spinning stage day-0, day-3), Ce (compound eye, mixed stages from 5th instar larva to pupa), Bs (Brain, mixed stages from day three 5th instar to S3), Bp (Brain, a mixture of pupal stages after P1, just after pupation), An (antenna, adult moth), Em (Embryo, 72 hours), Pb (whole mid-stage pupae without the cuticle). The number of EST is appended to the tissue type.

### OBP genes are clustered in the genome

**Figure 1:**
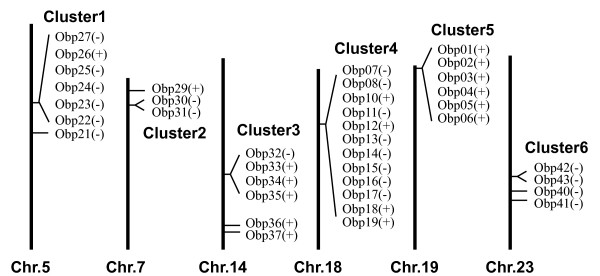
**Genomic locations of silkworm odorant-binding protein genes**. 38 OBP genes are distributed across six chromosomes. Another four OBP genes (obp20, obp38, 39 and 44) are represented on chromosome 26, 16, 22 and 12, respectively. The four genes have been omitted for clarity in Figure 1. BmOBP9 and BmOBP28 are not mapped to chromosomes. The horizontal lines represent the locations of each OBP gene. Transcriptional orientations of OBP genes are indicated by (+) or (-).

**Figure 2:**
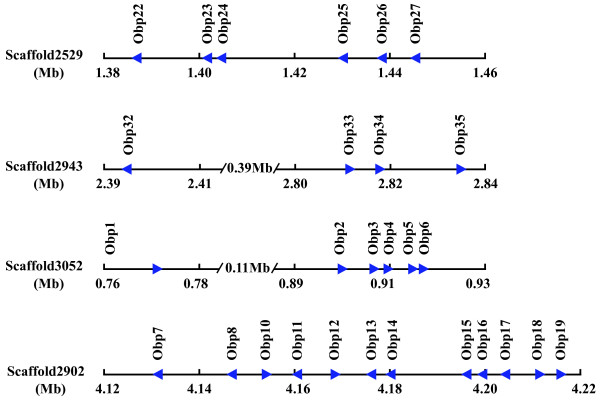
**Four representative OBP gene clusters present in silkworm**. Four gene clusters are located on scaffold2529, scaffold2902, scaffold2943 and scaffold3052, respectively. Each gene is depicted by arrowheads presenting the orientation of transcription in the scaffold. Three non-olfactory genes between *Obp1 *and *Obp2 *in scaffold3052 have been omitted for clarity.

42 OBP genes were distributed across 10 chromosomes, BmOBP28 is located on scaffold896, but cannot be mapped to a chromosome based on the current genome assembly (Figure 1, 2). More than three-quarters of the silkworm OBP genes are located within clusters as also seen in *D. melanogaster*, *A. gambiae *and *A. mellifera *[19,22,23]. Twenty OBP genes are organized into five clusters on five chromosomes. Of these gene clusters, only Cluster 5 includes intervening genes. The largest cluster (Cluster4) contains 12 OBP genes, which occur in both orientations within a 90 kb region on chromosome 18. Cluster5 (OBP1–6), including PBP1, GOBP1 and GOBP2 reported in previous studies, is located on chromosome 19 in the same orientation. Three non-OBP genes are located between OBP1 and OBP2. Cluster1 (OBP22–27) is located on chromosome 5. OBP26 is present in the reverse orientation to the other members of the cluster. Three smaller clusters (Cluster2, Cluster3 and Cluster6) containing two or four genes are present on chromosome 7, 14 and 23. This genome organization, and especially the presence of several large clusters, indicates a relatively recent expansion of the silkworm OBP family.

### Characteristics of the silkworm OBP family

**Figure 3:**
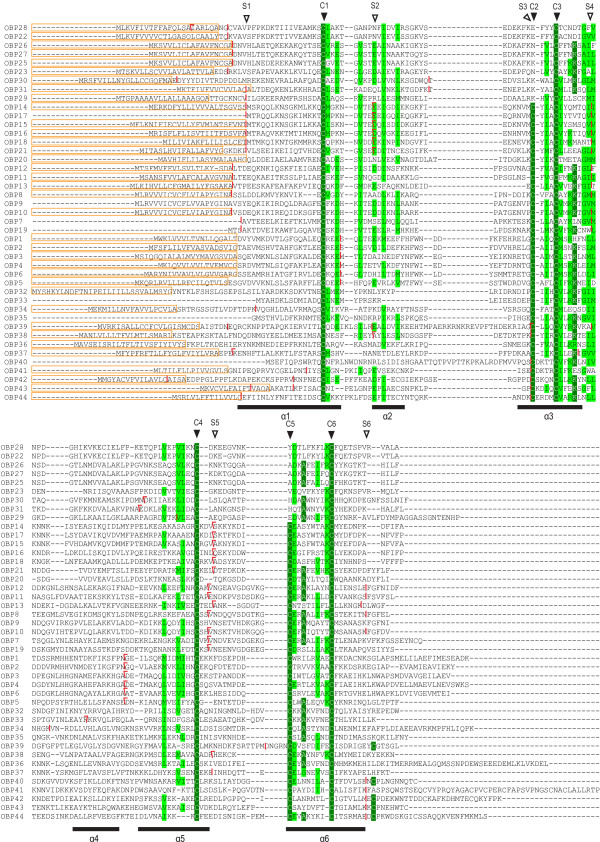
**Alignment of the silkworm OBP-like family members**. The signal peptides are boxed. Conserved residues are shown with a green or light green background. Highly conserved cysteine residues are marked by dark arrowheads. The rectangles under the alignment represent α-helices identified in BmorPBP1. Vertical bars indicate that the intron occurs between codons. Backward slanted separators and forward slanted separators point out the splice sites within codons after the first base and the second base, respectively. Six conservative splice sites are marked by hollow arrowheads.

Insect OBPs are generally quite divergent and the overall pairwise sequence identity is modest [19]. The alignment of the predicted silkworm OBP-like proteins (Figure 3) shows low average pairwise sequence identity between OBP family members. The predicted proteins have low molecular masses (14–22 kDa) and signal sequences are predicted at the hydrophobic N-terminus.

A six-cysteine signature is the most typical feature of classical insect OBPs [33]. The spacing pattern of conserved cysteines in the silkworm OBP family is similar to that in *Drosophila*. Following the naming system proposed by Hekmat-Scafe *et al*. [19], we refer to OBPs missing C2 and C5 as Minus-C OBPs, and those carrying more than six conserved cysteine residues as Plus-C OBPs. All six cysteine residues are completely conserved among twenty-nine typical silkworm OBPs. The spacing pattern of conserved cysteines in these typical OBPs is C1-X_25–68_-C2-X_3_-C3-X_31–46_-C4-X_8–29_-C5-X_8_-C6 (where X is any amino acid). There are ten Minus-C OBPs (OBP22–31) that are missing the second and the fifth cysteines. Five Plus-C OBP members (OBP40–44) carry additional conserved cysteines located between C1 and C2 and after C6.

The majority of silkworm OBP genes carry 0–4 introns that are located in conserved positions (Figure 3). Most introns are inserted in phase 0 and 1. Generally, the first intron is always present in phase 0, near the cleavage site of the predicted signal peptide. Six classes of conserved splice sites have been identified in the honey bee, *D. melanogaster*, *A. gambiae*, and *T castaneum *(Figure 3) [23]. The splice sites in most silkworm OBPs belong to one of the six classes. However, several genes appear to have introns inserted in nonconserved positions or phases, such as Cluster5 (Figure 3).

### Phylogenetic analysis of the silkworm OBP family

**Figure 4:**
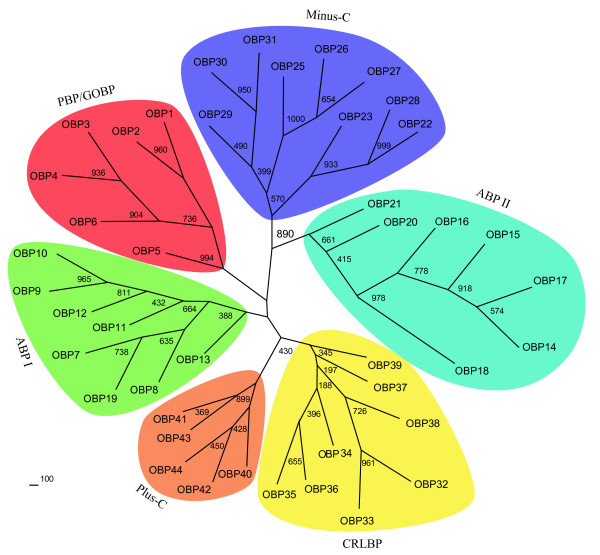
**Phylogenetic comparison of the OBP protein family members in the silkworm**. An unrooted distance (neighbor-joining) tree was constructed using an alignment of the silkworm OBP-like family members after removing the highly divergent signal peptide sequences at the N-terminus. Bootstrap support (1000 replications) is indicated at the major nodes.

The phylogenetic tree of the silkworm OBPs, constructed using the neighbor-joining method (Figure 4), indicates six possible protein subfamilies. Following the description by Hekmat-Scafe, we named these subfamilies as PBP/GOBP, CRLBP, ABPI and ABPII as well as the two atypical families Plus-C and Minus-C. High bootstrap values support many terminal relationships and three subfamilies: PBP/GOBP, Minus-C and ABPI. However there was weak support for the other three subfamilies: ABPII, Plus-C and CRLBP and the overall tree architecture. The groupings are supported by a number of additional features.

First, the spacing pattern of conserved cysteines is similar within each subfamily. The spacings of C1–C2 and C4–C5 in the PBP/GOBP and ABPI subfamilies are larger than in other subfamilies. By contrast, the spacing between C3 and C4 of Minus-C and ABPII is smaller than in the others. In all of the members of the Plus-C subfamily, C2 and C3 are separated by four residues, while C5 and C6 are separated by seven residues.

Second, the pairwise identity within each subfamily is higher than that between members of different subfamilies. The PBP/GOBP subfamily has the highest average pairwise sequence identity (36%), with a range from 22% to 55%. The average identities for the ABPII and Minus-C subfamilies are 35% and 29%, respectively. The other three subfamilies have lower internal sequence identities. Genes within the ABPI subfamily have lower average identity values than those in the ABPII subfamily.

Third, subfamilies are supported by the chromosomal clustering of OBP genes. The PBP/GOBP subfamily comprises the six members in Cluster 5. The Minus-C subfamily comprises nine members, of which seven occur in Cluster 1 and Cluster 2. OBP28, which is located on a small scaffold, shares high identity (78%) with OBP22. The Plus-C subfamily comprises five members, of which four members are located on chromosome 23. The CRLBP subfamily comprises eight members. Six of these are on chromosome 14 and *OBP32–35 *form Cluster 3. Gene Cluster 4 is divided into two subfamilies: the ABPI subfamily comprising seven members and the ABPII subfamily containing five genes. Two additional ABP family members are located on another scaffold. OBP20 has a single exon. In addition, we found the transcription terminating signal (AAACAAAA) in the 3' UTR. Two direct repeat sequences (TAATGAAATAAAATTA) are present in the 5'UTR and the 3'UTR. OBP20 may have moved to new genomic positions by retroposition.

Finally, all members within a subfamily share certain common intron insertion sites, which differ among subfamilies. The PBP/GOBP subfamily contains two intron insertion sites which were not found in *D. melanogaster*, *A. gambiae*, *A. mellifera *and *T. castaneum*. The ABPI and ABPII subfamilies have lost the conserved splice sites at S6 and S2, respectively. In the Minus-C subfamily, Cluster 1 and OBP28 have only one intron at the N-terminus, whereas Cluster 2 and OBP29 have additional introns at non-conserved sites. In the Plus-C subfamily, three common intron insertion sites are located at S1, S3 and S6 sites. In the CRLBP subfamily, OBP32, OBP35 and OBP36 have only one single exon each. OBP37 and OBP38 have conserved splice sites.

It is notable that the relatedness of the ABPII and Minus-C subfamilies are supported by a better bootstrap value than that for the ABPII and ABPI subfamilies. This interesting feature has also been found in bee and *Drosophila*. This suggests that the Minus-C subfamily may be derived from an ancestor with six conserved cysteines. To better understand the high degree of OBP sequence divergence, we analyzed the evolutionary constraints that were acting on this gene family. The average pairwise ratio of nonsynonymous to synonymous substitutions (*d*N/*d*S) for sequences in each subfamily was < 1. This indicates that there is strong negative selection for silkworm OBP genes. However, we observed that pairwise *d*N/*d*S values for several members of the Minus-C family are > 1 (see Additional file 1). This suggests that the members of the Minus-C subfamily are undergoing positive selection.

**Figure 5:**
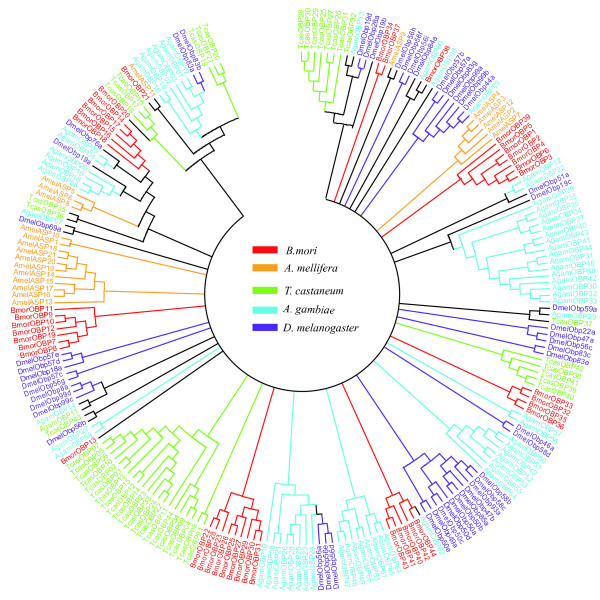
**A Neighbor-joining unrooted tree of annotated OBP protein among *B. mori, D. melanogaster, A. gambiae, T. castaneum and A. mellifera***. Bootstrap support is based on 1000 resampled data sets. The tree is condensed to show only branches with 15% bootstrap support or higher. Color of gene names indicates species.

We also built a phylogenetic tree based on the alignment of OBP sequences in five species, *B. mori, D. melanogaster, A. gambiae, T. castaneum *and *A. mellifera*, representing four orders (Figure 5). The six subfamilies for the silkworm defined above also form clades. Despite little bootstrap support for the clade, the Minus-C and ABP subfamilies are grouped together with OBPs in *A. mellifera *and *T. castaneum*. Only a few orthologies could be found among these five species (e.g., BmorOBP38 having two fly orthologues). The other subfamilies show no obvious relationship across species. This suggests that significant lineage-specific expansion and divergence have occurred in these insects.

### Expression patterns of the silkworm OBP genes

A numbers of EST libraries have been constructed for silkworm and more than 238,000 ESTs are available in GenBank. ESTs corresponding to 24 BmorOBP genes were identified using tBLASTn with BmorOBP protein sequences. The coding regions of 16 genes were covered completely by ESTs (Table 1). ESTs for 17 of the OBP genes were recovered broadly from chemosensory libraries, including larval maxillary galea, epidermis, brain and adult antennae. Interestingly, ESTs for seven of the OBP genes were only found in maxillary galea. OBP23 is present in silkgland and wing disk besides maxillary galea. It is noteworthy that OBP23 and OBP11 are highly expressed in maxillary galea, with 37 and 100 ESTs, respectively. Three OBP genes (OBP1–3), together with OBP14, are only observed in the antennal library. OBP27 is present in multiple tissues (silkgland, brain, malpighian tubule, fat body, midgut, wing disk and testis) as well as an antennal library. Three genes (OBP27, OBP30 and OBP31) were found in brain, with OBP31 represented by two ESTs in the compound eye. In larval epidermis, only one EST was found for OBP39. Meanwhile, ten BmorOBPs were recovered from non-chemosensory tissue libraries, such as silkgland, malpighian tubule, fat body, midgut, testis, ovary, compound eye, hemocyte, and wing disk. Most ESTs were also found in the fat body, ovary, testis, silkgland, and wing disk.

**Figure 6:**
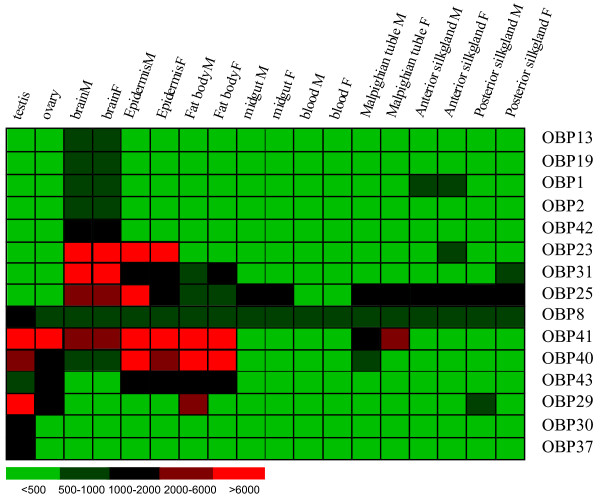
**Expression patterns of silkworm OBPs in multiple tissues of larvae on day 3 of the fifth instar**. The levels of expression are illustrated by a five grade color scale representing relative expression levels of < 500, 500–1000, 1000–2000, 2000–6000 and more than 6000.

We designed and constructed a genome-wide microarray using 70-mer oligonucleotides based on the draft silkworm genome sequence database. There are four probes, each of which contains a 20-bases stretch mismatching with their target genes. The probe sw11831 is for OBP7 and OBP8, and sw20121 is for OBP25–27. The gene expression patterns of OBPs were surveyed in multiple silkworm tissues on day 3 of the fifth instar and also in whole insects at 15 different time points from day 3 of the fifth instar larva through to the adult moth. A list of the thirty-one silkworm OBP probes used in this microarray is provided in Table 1. The expression data are visualized in Figure 6.

OBP gene expression in multiple tissues on day 3 of the fifth instar silkworm is consistent with EST representation in the database. We found fifteen genes with significant levels of expression (Figure 6). The expression profiles of OBP genes differ markedly even among members of the same gene cluster. The majority of OBPs are expressed in testis, ovary, brain, epidermis and fat body. Three OBPs (OBP23, 25 and 31) gave stronger signals in brain and epidermis than in other tissues. Five genes (OBP1, 2, 13, 19, and 42) are restricted to brain and have low expression levels. OBP40 and OBP41 share a similar expression pattern in six tissues. However, overall, OBP41 is expressed at higher levels than is OBP40. OBP43 is expressed at low levels in testis, ovary, epidermis and fat body, which is a different pattern than that for OBP42. Sex-biased expression was examined based on two-fold differences in expression level between the sexes. OBP29, which is expressed at the highest level in testis and at low levels in ovary and fat body of males, was the most interesting case. However, the expression of the majority of OBPs does not appear to have an obvious sexual bias on day 3 of the fifth instar.

**Figure 7:**
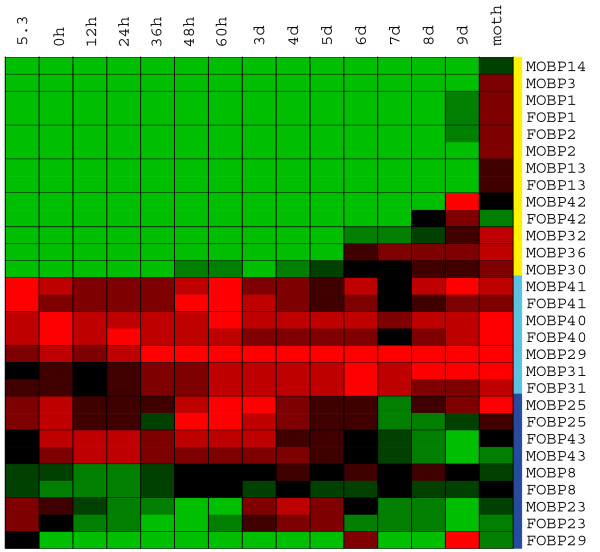
**The developmental expression patterns of silkworm OBPs**. The columns represents fifteen different sample time points: day three of the fifth instar and fourteen different times after spinning: 0 hour, 12 hours, 24 hours, 36 hours, 48 hours, 60 hours, 3 days, 4 days, 5 days, 6 days, 7 days, 8 days, 9 days and adult.

Our whole-organism array data failed to detect significant expression levels of 11 of the OBP genes at any time point in either sex. It is possible that some of these genes are expressed during these life stages but at levels below the detection limit. For example OBPs 13, 19 and 27, although not detected at the whole organism level in fifth instars, are detected in specific tissues at that life stage. Three classes of developmental expression profile for 18 members of the silkworm OBP-like family are shown in Figure 7. Expression of the first class of OBPs was restricted to adults and pupae near eclosion. A very faint signal was observed in male moths for OBP14. Three members of the same gene cluster (OBP1, 2, 3) and OBP13 were detected at moderate levels in adult. OBP3 was only observed in male. OBP42 was more abundant in males than females nine days after spinning. OBP30, OBP32 and OBP36 were detected with weak signals prior to the emergence and reached the highest level in male moths.

The second class of OBPs with three members (OBP31, 40, 41) is strongly expressed throughout all stages. Expression of OBP31 gradually rises and reaches its highest level in late pupae. OBP40 and OBP41 show three obvious expression peaks at larva, 60 h after spinning and adult. The expression of OBP29 in males, which is expressed at high levels in all lifes stages and reaches the highest level in adults, also follows this pattern.

Members of the third class of OBPs were expressed in several distinct phases. OBP23 is a good example with expression peaks in the larva, four days after spinning and again with a weak peak in the adult moth. The expression peaks of OBP25 are at 0 h, 60 h after spinning and in the adult. Furthermore, the transcripts were more abundant in males than in females at the late pupae stages. The highest expression level of OBP43 was at 12 h and gradually weakened until 6D after spinning. Only a weak signal was detected in the adult female. OBP8 shows a weak signal at several time points. OBP29 expression in females also followed this general pattern with high values 6d and 9d after spinning. This contrasts with its expression pattern in males, as described above.

**Figure 8:**
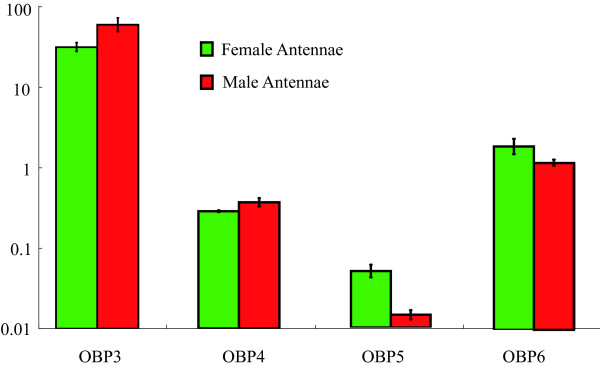
**Four candidate PBP gene expression levels in the antennae of moths**. Expression levels relative to the control gene *BmActin3 *are quantified by QRT-PCR. Bars on each column represent SD for three independent experiments.

In addition, we determined expression levels of OBP3–6 in moth antennae by QRT-PCR (Figure 8). Consistent with previous studies [32], BmorOBP3 was predominantly expressed in antennae of male moths. BmorOBP4 was equally expressed in antennae of both sexes. In contrast, BmorOBP5 showed substantially higher expression in female antennae. BmorOBP6 was marginally more highly expressed female antennae.

## Discussion

We have identified an OBP gene family comprising 44 members in the silkworm. This number is comparable to that in *D. melanogaster *and more than twice that in *A. mellifera*. Previously, eighteen chemosensory proteins (CSPs) have been identified in the silkworm genome [34]. Although there is no conclusive experimental support for olfactory functions, it was suggested that the CSPs may be a second class of OBPs. Moderate numbers of olfactory and gustatory receptors have been reported in the silkworm genome [35-37]. Recent evidence has demonstrated that OBPs are required for recognition of odorants or pheromones in a number of species [9-17]. The silkworm OBPs could play a chemosensory role in chemoreception by a combinatorial interaction with chemoreceptors.

Our phylogenetic analysis revealed that the silkworm OBP family comprises six subfamilies. Evidence of significant expansion of, and divergence among, OBP subfamilies indicates that the OBP family has undergone rapid evolution following a complex set of gene duplication events. This may have been required to enhance the ability to detect diverse sets of odorants. For example, silkworms at the larval stage can accurately distinguish volatile compounds and tastants released from mulberry leaves and other plants. The Plus-C OBP genes have only been found in silkworm, flies, and mosquitoes; therefore, we conclude that these genes arose after the separation of the Mecopteria. The honey bee possesses the smallest OBP repertoire. The silkworm ABP and Minus-C subfamilies share a high degree of sequence similarity to their homologues in honey bee. The divergence of the OBP families between silkworm and bee might be due to differences in their social organization, foraging behaviour and life cycles. Both species find food resources using plant volatiles, however, the range of food sources exploited by the honey bee and the sophistication of its chemical communication is substantially greater than for the silkworm. OBP subfamilies that are common to both species may be required for detection of similar odorants, such as plant volatiles. The silkworm-specific OBP subfamily may be important for oviposition, mate finding and so on. For example, the PBP\GOBP subfamily forms a monophyletic group specific for *Lepidoptera*.

The diversity of the OBP gene family suggests a role for positive selection in the rapid evolution and functional diversification of these genes. We found evidence for positive selection in the Minus-C OBP subfamily. Nevertheless, the relatively low dN/dS value in other OBP subfamilies suggests a purifying selection due to functional constraints. This conclusion is consistent with that obtained by investigating nucleotide variation in two OBPs (OS-E and OS-F) and comparative analysis of the OBP family in 12 *Drosophila *genomes [38-40]. Subfamily members arising by duplication may acquire subtle functional differences. Nevertheless, small changes in sequence may have profound functional consequences [41]. Moreover, the diversity of expression patterns and their expression as heterodimers or homodimers increase the potential function of the OBP family. Several studies have demonstrated that some OBPs might form homodimers or heterodimers [7,42-48].

The expression profiles of silkworm OBP genes have been characterized for both sexes and several tissues and a number of developmental stages. One group of OBP genes is expressed only in olfactory tissues, whereas others are expressed more broadly, including in tissues with no known olfactory function. The majority of members in the same subfamily show a similar expression pattern. In this study, we found that only members of the PBP/GOBP subfamily are antenna-specific. OBP1 and OBP2 were found to be expressed at approximately equal levels in the antennae of female and male moths by Northern blot [32]. The well characterized BmorPBP1 is predominantly expressed in the antennae of male moths. *In vitro *studies demonstrated that PBP1 could selectively bind the pheromone component bombykol [8,9]. The presence of four candidate BmorPBPs in the silkworm genome offers the possibility of selective transport of the three female pheromone components to receptor neurons [49,50]. This hypothesis is supported by binding studies with two PBPs from olfactory sensilla of the silkmoth species, *Antheraea polyphemus *and *Antheraea pernyi *[51]. Female moths release bombykal to repel potential mates. Based on the sequence conservation of the PBP gene family and the uniqueness of the PBP lineage in Lepidoptera, we speculate that BmorOBP4–6 may be candidate binding proteins for bombykal. In contrast to BmorOBP3, BmorOBP5 and BmorOBP6 seem to be expressed at higher levels in female moths. The two OBPs may bind other odors, including oviposition cues or as yet uncharacterized odors that may be released by male moths [52].

Another typical class of OBP is the ABP subfamily, most members of which are expressed specifically in chemosensory organs. Intriguingly, we found that OBP8–12 are expressed in larval maxillary galea which contains taste sensilla. Some *Drosophila *OBP genes are expressed in both olfactory and gustatory tissues and some are exclusively expressed in gustatory organs [18,53]. For example, *Drosophila *OBP57d and OBP57e are involved in taste perception and the response to toxins demonstrating that OBPs are important for gustation [15]. The ABPI subfamily in silkworm might be involved in the perception of the taste of mulberry leaves.

Several members of the Minus-C and Plus-C subfamilies are expressed in multiple tissues, including several non-chemosensory organs, such as fat body, testis, and ovary. Most of these are detectable throughout development and show three obvious peaks of expression. The expression patterns characteristic of this OBP subfamily are similar to those of the CSP family in silkworm and in other species. Although pupae are dormant, the transformation from larva to moth involves profound metabolic changes. Larval organs and appendages are digested internally and replaced by adult structures. The fat body supplies considerable energy for this metamorphosis. We hypothesize that some members of the OBP and CSP families may play general physiological roles as carriers or may mediate responses to ligands that are important for metamorphosis and development.

The silkworm OBP will assist the identification of OBPs from other insect species by similarity screening, especially in the Lepidoptera. The comparison of OBP families across many different insect species may shed light on evolutionary divergence among OBPs and on insect chemosensory mechanisms of host and environmental adaptation.

## Conclusion

44 OBPs have been identified in the genome of the silkworm. These may represent the entire repertoire of silkworm OBPs. Modest numbers of OBPs might interact with chemoreceptors to enhance the capabilities of chemoreception. The remarkable sequence divergence and subfamily expansion suggests that silkworm OBP family members bind to diverse sets of odorants. The family shows evidence of purifying selection, likely due to functional constraints. The expression profile of the OBP family suggests that these proteins might be involved in olfaction and gustation, as well as having general transport roles in non-chemosensory tissues.

## Methods

### Identification of the silkworm OBP family members

Known insect OBP sequences were downloaded from GenBank [19,22,23,54]. These insect OBP sequences were used to search for similar genes in the silkworm genome sequence with TBLASTN [55,56]. Silkworm genomic regions containing OBP genes were predicted using FGENESH+ [57]. Gene prediction was revised by comparing with the EST database. Candidate OBP genes were checked for three universal features of the insect OBP family: a conserved cysteine pattern, a predicted size (~14 kDa), and a signal sequence predicted by SignalP [58].

### Nomenclature of the silkworm OBPs

We adopted nomenclature for silkworm OBPs that is analogous to those proposed for the *Anopheles *OBP and the honey bee OBPs [22,23]. We use the prefix *BmorOBP *to reflect that the gene is a putative member belonging to the silkworm Odorant Binding Protein-like family. The previously published silkworm general binding protein (BmorGOBP) has been renamed BmorOBP1. OBP genes organized into a cluster were given consecutive numbers. The classical OBP members are listed prior to the atypical members (Table 1).

### Phylogenetic analysis

The forty-four conceptually translated protein sequences from silkworm OBP genes identified in this study, along with the OBPs from four other insect species (*D. melanogaster*, *A. gambiae*, *A. mellifera *and *T. castaneum*), were used to construct a phylogenetic tree. Sequences were aligned using ClustalX [59]. The α-helices identified in BmorPBP1 were indicated under the alignment [60]. The highly divergent signal peptide sequences at the N-terminus were truncated. Neighbor-joining trees were produced using the Phylip package [61]. Bootstrap analysis was performed using 1000 neighbor-joining replicates. The tree was displayed using MEGA4 [62]. All pairwise dn/ds values were calculated using the program KaKs_Calculator. The program adopts model selection and model averaging to calculate nonsynonymous (Ka) and synonymous (Ks) substitution rates [63]. In addition, several existing methods (NG, LWL, LPB, MLWL, MLPB, YN, MYN, and GY) for calculating Ka and Ks are also incorporated into KaKs_Calculator. Fisher's exact test for small samples was applied to justify the validity of Ka and Ks calculated.

### Sample preparation

The silkworm strain Dazao was reared on mulberry leaves and pupae were maintained at room temperature until eclosion. For the silkworm genome-wide oligonucleotide microarray, the anterior/median silk gland (A/MSG), posterior silk gland (PSG), testis, ovary, fat body, midgut, integument, hemocyte, malpighian tubule, and head on day 3 of the fifth instar were hand-dissected on ice. We also collected individuals at 15 different time points from day 3 of the fifth instar to moth. Moth antennae, heads, thoraces, abdomens, legs, wings were dissected and all tissues were immediately frozen with liquid nitrogen and stored at -70°C until use.

### Microarray analysis

A genome-wide microarray with 22,987 70-mer oligonucleotides was designed and constructed by Southwest University (Chongqing, China) and CapitalBio Corporation (Beijing, China) [64]. Briefly, the 70-mer probes representing 21,375 predicted genes from the silkworm WGS and 1,612 ESTs of interest that were not contained in the predicted genes were synthesized by MWG-Biotech Inc (Ebersberg, Germany). Arrays were fabricated using a SmartArrayer™ (CapitalBio Corporation, Beijing, China). cDNA labeled with fluorescent dye (Cy5 and Cy3-dCTP) was produced by the linear RNA amplification method [65]. The resulting labeled cDNAs were denatured in hybridization solution (3×SSC, 0.2% SDS, 5×Denhardt's solution and 25% formamide) at 95°C for 3 min before hybridization. Hybridizations were performed in a hybridization chamber which was placed in a three-phase tilting agitator (BioMixer™; CapitalBio). After hybridization, slides were washed twice with washing solution. We used a dual-dye experiment to analyze the expression patterns and each experiment was performed as a dye-swap. In pilot experiments, we performed several self-to-self hybridizations to evaluate system noise. All arrays were scanned with a confocal LuxScan™ scanner and the images obtained were analyzed using LuxScan™ 3.0 software (CapitalBio Corporation, Beijing, China). For the individual channel data extracts, we removed any faint spots from both channels (Cy3 and Cy5), whose signal intensities were below 400 units after subtracting the background. The linear normalization method was used to normalize individual channel data, based on the expression levels of four confirmed housekeeping genes. The normalized signal intensity values were further analyzed using one-way analysis of variance (ANOVA), with the significance level set at a P value of less than 0.001 (*P *< 0.001) across all investigated tissues. We visualized the cluster data using the Treeview program [66].

### QRT-PCR analysis

Total RNA was isolated from adult tissues using Trizol Reagent (Invitrogen). First-strand cDNA was synthesized using the superscript first-strand synthesis system for RT-PCR (Invitrogen). Gene-specific primers for RT-PCR were synthesized commercially and silkworm *actin3 *was used as an internal control. For OBP3, OBP4, OBP5 and OBP6, the QRT-PCR reactions were performed using an ABI PRISM™ 7000 Sequence Detection System (Applied Biosystems) and SYBR Green I (SYBR^® ^*Premix Ex Taq*™, TaKaRa). The PCR cycles were as follows: a 10 min denaturation at 95°C, followed by 40 cycles with 5s at 95°C and 30s at 60°C. The expression levels of the PBP candidate genes were calculated relative to the control gene *BmActin3 *according to Livak & Schmittgen [67].

## Authors' contributions

DPG carried out the analysis of genome and microarray data and drafted the manuscript. HJZ performed microarray analysis and revised the manuscript. PZ helped to perform the experiment of QRT-PCR experiment. QYX participated in conception and design of the study, and helped write the manuscript. ZHX contributed to the interpretation of results. All authors read and approved the final manuscript.

## Supplementary Material

Additional file 1 **Ka/Ks estimation based on model selection using KaKs_Calculator**. The data represent the average pairwise ratio of nonsynonymous to synonymous substitutions (*d*N/*d*S) for sequences in six subfamily. Click here for file
