# Supplementary material for: The Odorant Binding Protein Gene Family from the Genome of Silkworm, Bombyx mori
Source: BMC Genomics. 2009 Jul 23;10:332. doi: 10.1186/1471-2164-10-332 (PMC2722677; doi:10.1186/1471-2164-10-332)
Supplement: Additional file 1 — Ka/Ks estimation based on model selection using KaKs_Calculator. The data represent the average pairwise ratio of nonsynonymous to synonymous substitutions (dN/dS) for sequences in six subfamily. [file 1471-2164-10-332-S1.doc]

## Additional file 1

**Ka/Ks estimation based on model selection using KaKs_Calculator.**

| **OBP40** | **Plus-C subfamily** | | | |
| --- | --- | --- | --- | --- |
| **OBP41** | 0.54(0.84,1.55) |  |  |  |
| **OBP42** | 0.69(0.91,1.32) | 0.50(0.81,1.62) |  |  |
| **OBP43** | 0.85(0.96,1.12) | 0.44(0.78,1.77) | 0.46(0.79,1.71) |  |
| **OBP44** | 0.68(0.91,1.33) | 0.70(0.92,1.31) | 0.63(0.88,1.40) | 0.62(0.88,1.42) |

| **OBP1** | **PBP/GOBP subfamily** | | | | |
| --- | --- | --- | --- | --- | --- |
| **OBP2** | 0.16(0.46,2.92) |  |  |  |  |
| **OBP3** | 0.24(0.59,2.49) | 0.38(0.71,1.85) |  |  |  |
| **OBP4** | 0.31(0.66,2.15) | 0.27(0.61,2.23) | 0.11(0.34,3.19) |  |  |
| **OBP5** | 0.42(0.78,1.83) | 0.51(0.83,1.61) | 0.43(0.77,1.80) | 0.50(0.85,1.68) |  |
| **OBP6** | 0.31(0.66,2.16) | 0.31(0.63,2.04) | 0.20(0.50,2.51) | 0.18(0.46,2.58) | 0.61(0.86,1.41) |

| **OBP14** | **ABPⅡ subfamily** | | | | | |
| --- | --- | --- | --- | --- | --- | --- |
| **OBP15** | 0.07(0.26,3.85) |  |  |  |  |  |
| **OBP16** | 0.15(0.45,3.06) | 0.16(0.47,3.03) |  |  |  |  |
| **OBP17** | 0.04(0.18,4.25) | 0.05(0.19,4.12) | 0.12(0.38,3.15) |  |  |  |
| **OBP18** | 0.17(0.49,2.84) | 0.24(0.55,2.30) | 0.21(0.56,2.66) | 0.21(0.51,2.47) |  |  |
| **OBP20** | 0.65(0.89,1.37) | 0.97(0.99,1.02) | 0.78(0.93,1.20) | 0.78(0.93,1.20) | 0.40(0.74,1.86) |  |
| **OBP21** | 0.47(0.82,1.73) | 0.60(0.87,1.46) | 0.44(0.80,1.81) | 0.61(0.88,1.44) | 0.50(0.83,1.64) | 0.64(0.89,1.39) |

| **OBP32** | **CRLBP subfamily** | | | | | | |
| --- | --- | --- | --- | --- | --- | --- | --- |
| **OBP33** | 0.19(0.53,2.84) |  |  |  |  |  |  |
| **OBP34** | 0.66(0.90,1.37) | 0.63(0.89,1.40) |  |  |  |  |  |
| **OBP35** | 0.56(0.88,1.57) | 0.66(0.90,1.38) | 0.52(0.82,1.57) |  |  |  |  |
| **OBP36** | 0.86(0.97,1.12) | 0.78(0.94,1.20) | 0.55(0.84,1.53) | 0.42(0.78,1.86) |  |  |  |
| **OBP37** | 0.76(0.94,1.24) | 0.65(0.90,1.39) | 0.75(0.93,1.25) | 0.70(0.92,1.31) | 0.70(0.92,1.31) |  |  |
| **OBP38** | 0.73(0.93,1.28) | 0.59(0.88,1.50) | 0.74(0.92,1.25) | 1.09(1.02,0.93) | 0.95(0.99,1.04) | 0.78(0.94,1.21) |  |
| **OBP39** | 0.93(0.98,1.06) | 0.84(0.96,1.14) | 0.82(0.95,1.16) | 0.79(0.95,1.20) | 0.91(0.98,1.08) | 0.83(0.96,1.16) | 0.71(0.92,1.30) |

| **OBP7** | **ABPⅠ subfamily** | | | | | | |
| --- | --- | --- | --- | --- | --- | --- | --- |
| **OBP8** | 0.32(0.69,2.15) |  |  |  |  |  |  |
| **OBP9** | 0.63(0.87,1.38) | 0.29(0.65,2.27) |  |  |  |  |  |
| **OBP10** | 0.55(0.83,1.52) | 0.23(0.59,2.56) | 0.55(0.05,0.09) |  |  |  |  |
| **OBP11** | 0.47(0.80,1.70) | 0.49(0.80,1.64) | 0.48(0.80,1.66) | 0.50(0.81,1.63) |  |  |  |
| **OBP12** | 0.35(0.72,2.05) | 0.44(0.75,1.70) | 0.35(0.65,1.86) | 0.32(0.63,1.98) | 0.37(0.72,1.93) |  |  |
| **OBP13** | 0.64(0.90,1.40) | 0.48(0.80,1.69) | 0.60(0.87,1.45) | 0.62(0.89,1.42) | 0.46(0.78,1.71) | 0.58(0.87,1.50) |  |
| **OBP19** | 0.40(0.74,1.85) | 0.46(0.81,1.74) | 0.57(0.86,1.52) | 0.53(0.85,1.59) | 0.48(0.80,1.66) | 0.34(0.71,2.12) | 0.71(0.92,1.30) |

| **OBP22** | **Minus-C subfaimly** | | | | | | | |
| --- | --- | --- | --- | --- | --- | --- | --- | --- |
| **OBP23** | 0.52(0.81,1.57) |  |  |  |  |  |  |  |
| **OBP25** | 0.51(0.82,1.59) | 0.56(0.82,1.48) |  |  |  |  |  |  |
| **OBP26** | 0.50(0.81,1.62) | 0.55(0.82,1.49) | 1.54(0.10,0.06) |  |  |  |  |  |
| **OBP27** | 0.54(0.83,1.54) | 0.55(0.82,1.49) | 2.05(0.08,0.04) | 1.15(0.04,0.04) |  |  |  |  |
| **OBP28** | 0.84(0.17,0.20) | 0.42(0.75,1.78) | 0.49(0.80,1.65) | 0.49(0.79,1.63) | 0.53(0.81,1.55) |  |  |  |
| **OBP29** | 0.69(0.90,1.31) | 0.62(0.87,1.42) | 0.56(0.83,1.48) | 0.56(0.84,1.49) | 0.54(0.83,1.52) | 0.71(0.91,1.28) |  |  |
| **OBP30** | 0.64(0.88,1.38) | 0.62(0.87,1.41) | 0.57(0.86,1.50) | 0.61(0.88,1.45) | 0.59(0.87,1.47) | 0.61(0.87,1.44) | 0.51(0.82,1.61) |  |
| **OBP31** | 0.59(0.87,1.46) | 0.50(0.82,1.64) | 0.36(0.72,1.99) | 0.36(0.72,2.00) | 0.33(0.71,2.16) | 0.53(0.84,1.57) | 0.42(0.78,1.87) | 0.19(0.49,2.66) |
